# Ubiquitination in the regulation of autophagy

**DOI:** 10.3724/abbs.2023149

**Published:** 2023-08-16

**Authors:** Xueyan Cen, Ziling Li, Xinpeng Chen

**Affiliations:** 1 Hubei Key laboratory of Edible Wild Plants Conservation & Utilization Hubei Engineering Research Center of Special Wild Vegetables Breeding and Comprehensive Utilization Technology School of Life Science Hubei Normal University Huangshi 435002 China; 2 National Laboratory of Biomacromolecules CAS Center for Excellence in Biomacromolecules Institute of Biophysics Chinese Academy of Sciences Beijing 100101 China

**Keywords:** monoubiquitination, polyubiquitination, autophagy molecule machine, selective autophagy

## Abstract

Autophagy, an efficient and effective approach to clear rapidly damaged organelles, macromolecules, and other harmful cellular components, enables the recycling of nutrient materials and supply of nutrients to maintain cellular homeostasis. Ubiquitination plays an important regulatory role in autophagy. This paper summarizes the most recent progress in ubiquitin modification in various stages of autophagy, including initiation, elongation, and termination. Moreover, this paper shows that ubiquitination is an important way through which selective autophagy achieves substrate specificity. Furthermore, we note the distinction between monoubiquitination and polyubiquitination in the regulation of autophagy. Compared with monoubiquitination, polyubiquitination is a more common strategy to regulate the activity of the autophagy molecular machinery. In addition, the role of ubiquitination in the closure and fusion of autophagosomes warrants further study. This article not only clarifies the regulatory mechanism of autophagy but also contributes to a deeper understanding of the importance of ubiquitination modification.

## Introduction

**Figure 1:**
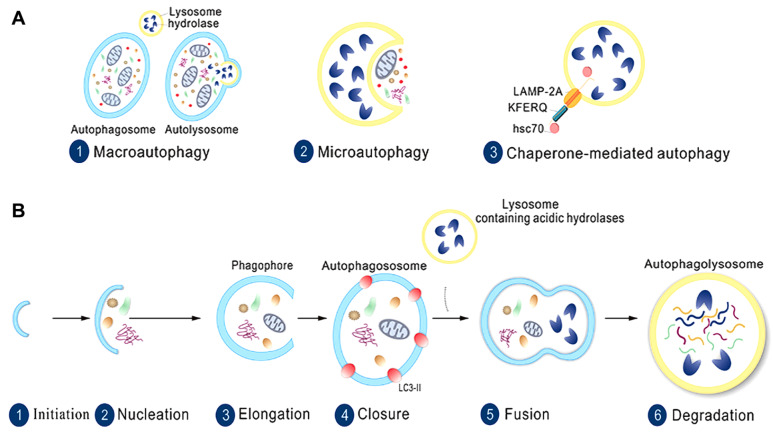
Figure 1 The types and stages of autophagy (A) Three types of autophagy: macroautophagy, microautophagy, and chaperone-mediated autophagy. (B) The stages of autophagy are divided into six steps: initiation, nucleation, elongation, closure, fusion, and degradation.

Autophagy is a crucial physiological activity that involves the transport of substances within vesicles to lysosomes for degradation, which maintains cell metabolism homeostasis. According to the specific mode of transporting goods, there are three types of autophagy, namely, macroautophagy, microautophagy, and chaperone-mediated autophagy ( Figure 1A).

The process of autophagy is believed to be divided into a series of successive stages: initiation, elongation, maturation, and fusion. Once autophagy is triggered, cytoplasmic goods are recruited to the isolation membrane termed the phagophore. The phagophore gradually extends and closes to form a double-membrane organelle named the autophagosome. Then, the autophagosome is delivered by some molecular motors, and its outer membrane is fused with the lysosomal membrane, leading to the release of enclosed cargos of the autophagosome to be digested ( Figure 1B). When exposed to acute stimulation (typically nutrition deprivation), autophagy is rapidly activated to cope with environmental change [1]. Many autophagy-related genes (ATGs) were identified by genetic screening in yeast [2], and these *ATG* genes are highly conserved with many mammalian genes. To date, over 40 *ATG* genes have been cloned; these genes act in concert with many complexes to execute autophagic events [3]. Because cytoplasmic cargos can be randomly packed into a phagophore, autophagy was once considered the nonselective degradation of goods. However, it has since been discovered that autophagy also selectively degrades cargo such as damaged mitochondria (mitophagy) and infected bacteria (xenophagy) [4]. There are also ribophagy and proteaphagy; for example, NUFIP1 (nuclear FMR1-interacting protein 1) was identified as a selective ribophagy receptor in mammalian cells. Therefore, selective autophagy controls the quality of many organelles and proteins. Each step of autophagy is precisely controlled by many protein complexes, and it is essential to understand how these complexes regulate and highly coordinate to carry out the process.

Ubiquitination, one of the posttranslational modifications on proteins, acts as a common strategy to modulate the activity of proteins because this modification endows proteins with structural diversity [5]. During autophagy, many cargos and ATG proteins are attached to ubiquitin, which enables modulation of protein location, distribution, and activity [ 6, 7].

Here, we review the advances in understanding the role of ubiquitination in the regulation of autophagy.

## Two Types of Ubiquitination

Ubiquitin, a compact globular protein consisting of 76 amino acids, exists widely in all eukaryotes [8]. Ubiquitination is the process by which ubiquitin is covalently conjugated to a given amino acid (usually lysine) on the target protein under the successive actions of ubiquitin-activating enzyme (E1), ubiquitin-conjugating enzyme (E2), and ubiquitin-ligating enzyme (E3) [ 8– 10]. Ubiquitin is initially attached to E1 in an ATP-dependent manner. Subsequently, it is activated and transmitted to E2. Next, E2 also binds to ubiquitin and transfers ubiquitin to E3. The last step involves the formation of the isopeptide bond between the ε-amino group on the lysine residue of the target protein and the C-terminal Gly carboxyl group of ubiquitin, and E3 ultimately catalyzes the particular combination of ubiquitin and the target protein with an isopeptide bond [ 1, 4]. In addition to the lysine residue, the C-terminus of ubiquitin can be optionally linked to Met1 on the N-terminus of the target protein [ 11, 12] .

**Figure 2:**
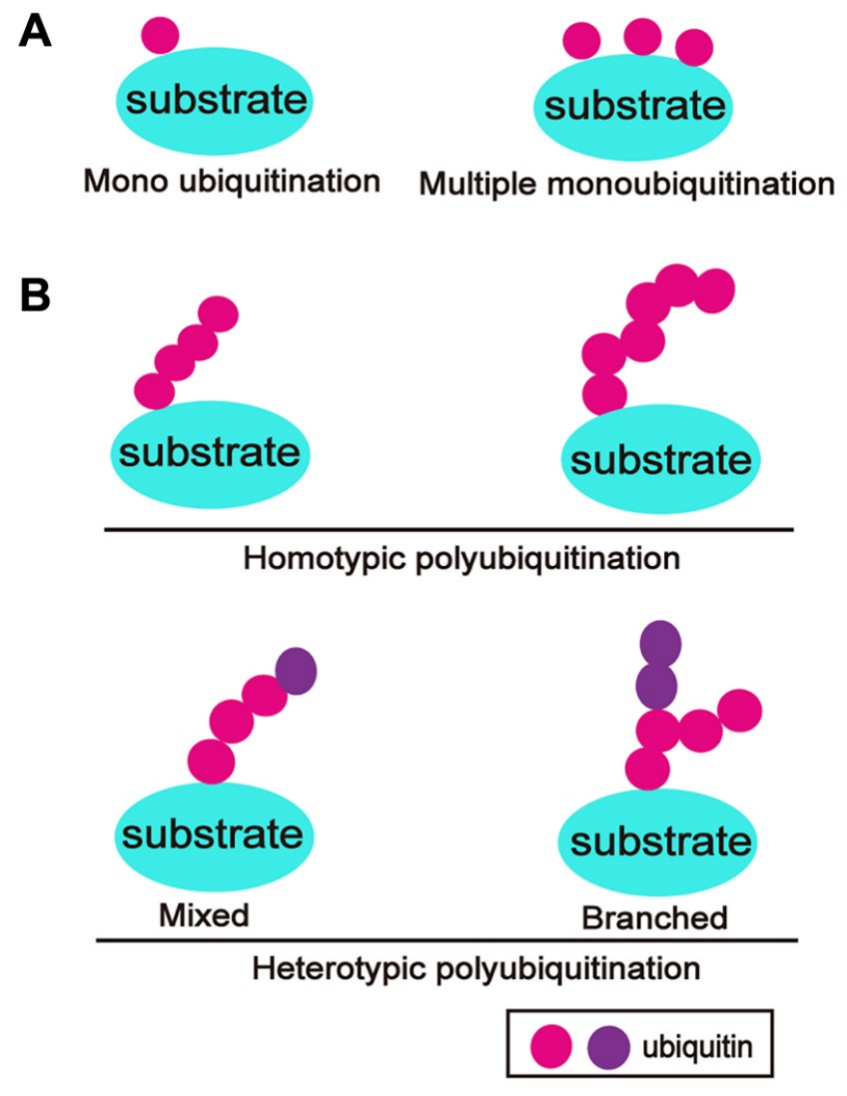
Figure 2 Ubiquitination diversity (A) Mono-ubiquitination occurs at a single site or multiple sites on substrates. (B) Polyubiquitination: homo-ubiquitination and hetero-ubiquitination. Homo-ubiquitination is the process by which ubiquitin is linked to its same lysine residue or methionine residue. Ubiquitin is linked to different lysine residues, which is the definition of hetero-ubiquitination.

Target proteins can be ubiquitinated in two ways, monoubiquitination and polyubiquitination [13]. Monoubiquitination is the simplest modification, in which a single ubiquitin molecule is covalently integrated to a Lys residue on a target protein or multiple ubiquitins are linked to different lysine residues [14] ( Figure 2A). There are two categories of polyubiquitination: homopolyubiquitination and heteropolyubiquitination. Homodiubiquitination is the process by which ubiquitin molecules are linked in just one manner, such as head-to-tail, to seven Lys residues (K6, K11, K27, K29, K33, K48, K63) on ubiquitin, which creates seven distinct ubiquitin chains [8]. In contrast, ubiquitin molecules form mixed or branched ubiquitin chains, described as heteropolyubiquitination, when they attach to specific amino acids on the target proteins [ 15 , 16] ( Figure 2B).

## The Role of Monoubiquitination and Polyubiquitination in Autophagy

### Ubiquitination in autophagy initiation

The formation of an autophagosome is the first step of autophagy. The event is orchestrated by three main complexes, including the serine/threonine protein kinase ULK1 (unc-51-like autophagy activating kinase 1) complex, the VPS (vacuolar protein sorting) 34 (VPS34) complex, and the ATG16L1-ATG5-ATG12 complex. ULK1, homologous to yeast ATG1, is a core component of its complexes that consist of three partners: FIP200 (focal adhesion kinase family interacting protein of 200 kDa), ATG13, and ATG101 [17]. Upon induction of autophagy, the activated ULK1 complex subsequently catalyzes the second kinase complex, VPS34, which is responsible for the production of phospholipid phosphatidylinositol 3-phosphate (PI3P) at the site of the phagophore. The VPS34 complex is composed of the class III phosphatidylinositol 3-kinase VPS34, Beclin-1, VPS15, and ATG14L (ATG14-like). The third complex acts as a conjugation system to join the ubiquitin-like ATG8 family (LC3, microtubule-associated protein 1 light chain 3) together to its target cargos [18].

**Figure 3:**
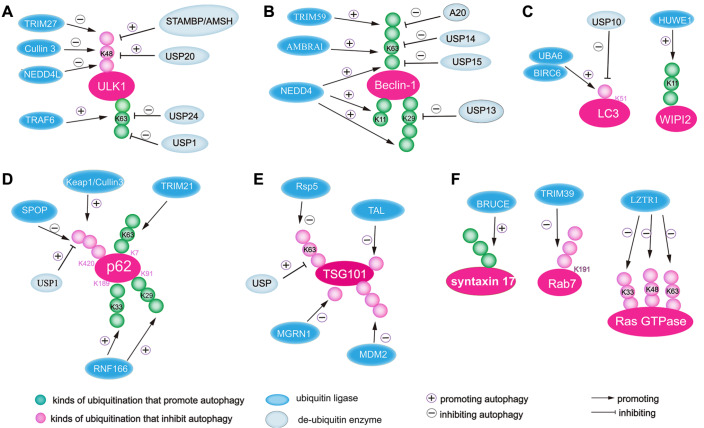
Figure 3 Ubiquitination is involved in the regulation of autophagy (A) The K48-linked ubiquitin chain destabilizes ULK1 to terminate autophagy, while the K63-linked ubiquitin chain triggers ULK1 activity to promote autophagy. (B) Beclin-1 decorated with the K63-linked ubiquitin chain promotes autophagy. The modification is balanced by ubiquitin ligases and de-ubiquitination enzymes. (C) During the formation of the autophagosome, LC3 is tagged with a mono-ubiquitination signal, while WIPI2 is polyubiquitinated. (D) During the assembly of cargo, p62 is labeled with K63-linked ubiquitin chains at different sites. (E) During autophagosome closure, the core component of the ESCRT complex, TSG101 is mono- or poly-ubiquitinated to cause degradation. (F) Small GTPase is likely to be linked to different polyubiquitin chains, which inhibits the fusion between autophagosome and lysosome. Syntaxin 17 with polyubiquitin chains promotes the fusion of the autophagosome and lysosome.

ULK1 activity is triggered after phosphorylation at different sites by several upstream kinase complexes, including AMP-activated protein kinase (AMPK) and MAPK1/3 kinase, after nutrient deprivation [ 19, 20] but is prevented by the mammalian target of rapamycin (mTOR) under abundant nutrient supply [21]. To date, many studies have shed light on the effect of ubiquitination on the activity and stability of ULK1. Upon autophagy induction, ULK1 autophosphorylation facilitates the formation of autophagosomes. The time and duration of autophagy depend on the nutrient supply. Once cells have received an abundant supply of nutrients, autophagy will be downregulated. ULK1 is reported to be a substrate of the ubiquitin ligase Cullin 3 and decorated with K48-linked polyubiquitination when cells are starved by culturing in Earle’s balanced salt solution. K48-linked polyubiquitination of ULK1 is a signal for proteasomal degradation. Downregulation of ULK1 reduces autophagy activity. KLHL20 is a substrate adaptor of Cullin 3 ubiquitin ligase. When KLHL20 is depleted, ULK1 ubiquitination is completely abrogated, and autophagy termination is significantly disrupted. Moreover, KLHL20 also associates with two autophagy initiation proteins, Beclin-1 and VPS34. Polyubiquitination mediated by KLHL20 promotes their turnover and accelerates the termination of autophagy [22]. Consistent with this finding, both E3 ubiquitin ligase TRIM27 (transmembrane protein 27) and NEDD4L (Nedd4 Like) also function as negative regulatory components of the FIP200-ATG13-ULK1 complex [23]. These results support that ubiquitination suppresses autophagy initiation via the ubiquitin proteosome system (UPS) to downregulate the initiation factor ULK1. TRIM27 adds the K48-linked polyubiquitination chain to the two sites of ULK1 and tags nondegradative K6- and K11-linked ubiquitination on serine/threonine kinase 38-like (STK38L) kinase, which is responsible for autophosphorylation of ULK1. Interestingly, the ubiquitination of STK38L enhances its activity and further increases the ubiquitination of ULK1. In turn, hyperubiquitinated ULK1 is transported to the proteasome for degradation. This leads to reduced ULK1 and retraining autophagy initiation [24]. Contrary to degraded signaling K48-linked ubiquitination, ULK1 is decorated with K63-linked polyubiquitination by ubiquitin ligase TRAF6 (tumor necrosis receptor-associated factor 6), which supports its stabilization and activity. AMBRA1, as a substrate of ULK1, is dephosphorylated and recruits TRAF6 to enhance ULK1 activity upon starvation [25]. However, transmembrane protein 189 (TMEM189) disrupts the interaction between TRAF6 and ULK1, leading to K63-linked polyubiquitination of ULK1 and increasing its instability. Therefore, ULK1 labelled with K63-linked polyubiquitination is proposed to play a positive role in priming the autophagosome [26]. In contrast to these ubiquitin ligases, deubiquitin enzymes (DUBs) are also involved in autophagy initiation by modulating the ubiquitination of ULK1. High-throughput screening assays of functional DUBs in autophagy have been performed, and several DUBs are candidates for targeting ATG proteins. It has also been shown that STAMBP/AMSH (STAM-binding protein) stabilizes ULK1 by removing its K48-linked ubiquitin chains and then initializes autophagosome formation [27]. To identify the DUB candidates that maintain the stability of ULK1, loss-of-function screens of DUBs were performed in HeLa cells using siRNA libraries targeting 99 DUBs. The results show that ubiquitin-specific peptidase 20 (USP20) interacts with and removes the ULK1 ubiquitin moiety to stabilize ULK1 under normal conditions, while USP20 dissociates from ULK1 at a later stage of autophagy [28]. Unlike USP20, ULK1 ubiquitination blocks autophagic flux after starvation and is attenuated by two deubiquitinating enzymes, USP1 and USP24. Previous reports support that ULK1 tagged with the K63-linked ubiquitination chain increases its stability, which suggests that the two DUBs could remove the K63-linked ubiquitin chain [ 29, 30] ( Figure 3A). In addition to ULK1, ubiquitination is an important strategy to regulate the turnover of two other components of the ULK1 complex, namely, FIP200/BRCC1 and ATG13, to drive autophagy initiation via UPS. FIP200/BRCC1 and ATG13 are also polyubiquitinated by different E3 ubiquitin ligases, and their downregulation impairs autophagy [ 31, 32].

The VSP34 complex, the second crucial complex that also controls autophagy initiation, is phosphorylated by ULK1 to induce its activity. Beclin-1 is the core component of the VSP34 complex and was originally identified in yeast. The dual role of Beclin-1 in autophagy and apoptosis in mammals has been well studied [33]. Beclin-1 consists of four domains: the N-terminal Bcl2 homology (BH)-3 domain, a central coiled-coil domain (CCD), an evolutionarily conserved domain (ECD), and an overlapping C-terminal β-α-repeated, autophagy-specific (BARA) domain [34]. Beclin-1 together with both VPS34 and VPS15 forms a highly regulated complex to promote local PtdIns-3P (phosphatidylinositol 3-phosphate) generation to prime autophagy [35]. In addition to phosphorylation mediated by the kinase ULK1, ubiquitination regulates the activity and stability of Beclin-1. These ubiquitin ligases link different types of polyubiquitin chains to different sites of Beclin-1, and these sites have also been mapped [ 36– 39]. Typical K63-linked ubiquitination of Beclin-1 is a nondegraded signal and blocks its association with Bcl2. Bcl2 is the founding member of the Bcl-2 family of regulator proteins that restrain cell death, which is required for autophagy induction [36]. The signal is strengthened by two ubiquitin ligases, AMBRA1 and tripartite motif protein 59 (TRIM59). TRIM59 can label both autophagy cargo receptors and platforms assembling autophagosome-formation machinery with ubiquitin [ 37, 40]. In addition to the ubiquitin ligase AMBRA1, some deubiquitination enzymes also play an important role: for example, in regulating the activity of VPS34, NEDD4/NEDD4-1 undergoes K29-linked autoubiquitination at K1279, recruiting USP13 to PIK3C3/VPS34, which reduces the K48-linked ubiquitination of PIK3C3/VPS34 at K419 and promotes stabilization [41]. Additionally, mixed K63- and K11-linked polyubiquitination also increases Beclin-1 stability. The ubiquitin ligase Nedd4 (neural-precursor-cell-expressed developmentally downregulated 4) ligates Beclin-1 with K11- and K63-linked chains, and the depletion of the Beclin-1-interacting protein VPS34 causes Nedd4-mediated proteasomal degradation of Beclin-1 via K11-linked polyubiquitin chains [38]. This indicates that VSP34 might have a positive effect on the ubiquitination of Beclin-1. However, the nondegradative K63-linked ubiquitination of Beclin-1 is counteracted by many deubiquitinating enzymes, including A20, USP15, and USP14 [ 42, 43]. USP14 activity is suppressed after phosphorylation by protein kinase B (AKT). It decreased the K63-linked ubiquitination of Beclin-1 and had a negative effect on autophagy [43] ( Figure 3B).

### Ubiquitination of LC3 conjugation machinery

Microtubule-associated protein 1 light chain 3B (LC3), encoded by the *MAP1LC3B* gene, primarily plays a role in cargo recruitment and autophagosome formation and is commonly used as an autophagosome marker [44]. LC3 is homologous to yeast ATG8 and shares an LDS (LIR docking site) hydrophobic binding interface in the N-terminus. This domain helps LC3 interact with LIR (LC3 interacting region)-containing proteins. These LIR-containing proteins are usually cargo-receptor proteins that associate with degraded cargo. Nascent soluble LC3 is cleaved at the C-terminal (LC3-I) and then conjugated to the head group of the lipid phosphatidylethanolamine by the ATG16L1-ATG5-ATG12 complex [45]. The lipidated form of LC3, termed LC3II, inserts in the membrane of the phagophore, and its N-terminus facilitates receptor-cargo recruitment. This lipidation conversion of LC3 is catalyzed by the autophagy initiation complex [ 46, 47]. Moreover, LC3 has been identified as an RNA-binding protein that triggers rapid mRNA degradation during autophagy [48].

Deacetylation was reported to drive LC3 transport from the nucleus to the cytoplasm during autophagy initiation [49]. Originally, the E3 ubiquitin ligase Nedd4 was considered to interact with LC3. Depletion of Nedd4 dramatically reduced the LC3 protein level and the number of autophagosomes. However, unexpectedly, LC3 is not the substrate of the ubiquitin ligase Nedd4 [50]. Recently, Jia *et al*. [51] performed a genome-wide CRISPR-Cas9 knockout screen assay to identify the ubiquitin ligase targeting LC3 in H4 human neuroglioma cells expressing endogenous LC3B tagged with GFP-mCherry as a reporter. They identified that ubiquitin ligase BIRC6 cooperates with ubiquitin-activating enzyme UBA6 to monoubiquitinate—rather than polyubiquitinate—K51 of LC3B, promoting LC3 degradation in the proteasomal pathway. UBA6 tags the monoubiquitination of LC3I without reducing the LC3II level [51]. Interestingly, approximately half of all proteins are monoubiquitinated or multimonoubiquitinated and then degraded by proteasomes in human cells, especially small proteins with 120–150 aa residues [52]. In contrast to the finding that acetylation promotes LC3 stability [ 53, 54], LC3 monoubiquitination is the signal for degradation after nutrient deprivation. However, acetylated LC3 acts as a nonactivated form and is suitable for storage. When nutrition is deprived, LC3 is deacetylated in the nucleus and readily binds cargo receptor p62. Interestingly, removing monoubiquitinated LC3 increased the level of LC3I but not activated LC3II. Therefore, reducing LC3 monoubiquitination promotes autophagic flux after nutrient starvation. Monoubiquitination also occurs in the nonactivated form of LC3I and shares the same amino acid residue with acetylation modification. It remains unknown whether the monoubiquitination of LC3 suppresses LC3 acetylation. The deubiquitination enzyme also participates in the stability of LC3. Recently, it has been reported that the mono-ubiquitin moiety of LC3 is removed by USP10 during starvation, resulting in suppression of the degradation of LC3 [55].

In addition to LC3 stability, LC3 lipidation is a prerequisite for the formation of autophagosomes. WIPI2 (WD40-repeat-containing PI3P-binding protein) facilitates LC3 lipidation and extends the phagophore by recruiting the Atg12-Atg5-Atg16L1 complex [45]. WIPI2 is inactivated by mTORC1 and polyubiquitinated by the E3 ubiquitin ligase HUWE1. During autophagy induction, the WIPI2 protein level is rapidly upregulated to promote LC3 lipidation [56] ( Figure 3C).

### Ubiquitination of autophagic cargo receptors

Autophagic cargo receptors are a group of proteins that serve as a bridge between lipidated ATG8 family proteins and degraded cargos attached to ubiquitin tags, which facilitates autophagosome formation. A growing number of studies in past decades have elucidated how autophagic cargo receptors selectively engulf their substrates. These common receptors are characterized by LC3-interacting region (LIR) motifs that directly or indirectly bind to the LDS of ATG8 proteins (LC3) and the ubiquitin-binding domain (UBD) [57]. Recently, some novel receptors have been discovered by quantitative proteomics methods; these receptors are independent of ubiquitin-binding receptors [ 58 – 60].

p62 (sequestosome-1) and NBR1 are two classical cargo receptors that recognize their cargos, which include damaged organelles or misfolded protein aggregates in mammalian cells [61]. Misfolded proteins are conjugated with polyubiquitin chains and recognized by p62 before autophagosomal maturation. In this process, p62 promotes the condensation of ubiquitinated proteins with the help of FIP200 to form a large structure and links the cargo to the nascent autophagosomal membrane via its interaction with LC3 [ 62 , 63]. In contrast, p62 oligomerizes into filaments to reinforce its affinity for LC3, which decorates the autophagosomal membrane [64]. In this process, both oligomerization and adaptors of p62 are pivotal to autophagosome maturity. Ubiquitin ligase Keap1/Cullin 3 was previously reported to ubiquitinate p62 at K420 within its UBA domain and induce p62 proteasomal degradation. The K420 mutation of p62 abolishes its sequestration and inhibits its degradation [65]. In contrast to the ubiquitin-proteosome degradation of p62 mediated by Keap1/Cullin3, SPOP is an E3 ubiquitin ligase, and its mutation contributes to prostate tumorigenesis. It binds to cytoplasmic p62 and then induces polyubiquitination of p62 at the K420 residue. This modification disrupts the self-association of p62 and liquid phase condensation, thus relieving p62-mediated Keap1 sequestration [66]. The finding that ubiquitination inhibits p62 oligomerization is further supported by another group [ 67 , 68]. Although the two ubiquitin ligases share the same site of ubiquitination of p62 at K420, the results seem to be contradictory. In addition to ubiquitination of p62 at K420, tripartite motif-containing protein 21 (TRIM21) is an E3 ubiquitin ligase that decorates the K7 site of p62 with K63-linked polyubiquitination. It plays an important role in autophagy and innate immunity. IFN-β increases TRIM21 activity for K63-linkage-specific ubiquitination, which prevents its self-oligomerization and targeting of the autophagosome [67]. Moreover, ubiquitination regulates the interaction of p62 with degraded cargos. When cells are infected with pathogens, the number of pathogens is decreased by the recruitment of ubiquitin as well as the autophagy adaptor p62. The E3 ubiquitin ligase RNF166 induces K29- and K33-linked polyubiquitination of p62 at residues K91 and K189, respectively. The two modifications enhance the affinity between p62 and the pathogen, which accelerates the clearance of pathogens. Therefore, p62 ubiquitination limits pathogen replication [69]. In addition to ubiquitin ligases, the deubiquitinating enzyme USP8 inhibits this decoration of p62 at the K420 residue and acts as a negative regulator of autophagy ( Figure 3D). USP8 interacts with and preferentially deubiquitinates the K11-linked ubiquitin chains of p62, which suppresses the degradation of p62 [70].

Another cargo receptor, NBR1, shares a similar ability with p62 and consists of three domains, namely, PB1, LIR, and UBD. NBR1 can also bind to both LC3 and ubiquitin. However, NBR1 cannot polymerize without the PB1 domain, but it needs to cooperate with p62 to form a polymeric chain [71].

### Ubiquitination in autophagosome closure

Although autophagy involves several steps, including phagophore assembly structure formation, expansion, and autophagosome closure and fusion, autophagosome closure has drawn little attention and is the least well-characterized event. The process of cellular membrane scission is mediated partly by the assembly of endosomal sorting complex required for transport (ESCRT) machinery proteins in a ring at the aperture of the closing phagophore [72]. ESCRT was originally identified as a subgroup of *VPS* genes in yeast and then found to mediate various topologically related membrane scission events [ 73, 74]. In the schematic model of autophagosome closure mediated by ESCRT-I and ESCRT-III, the small GTPase Rab5 promotes the interaction between Atg17 and ESCRT subunits; then, ESCRT-I is recruited to localize on the membrane of the autophagosome. Finally, ESCRT-III finishes constricting the membrane rim and mediating membrane scission [ 72, 75, 76].

The ESCRT-I complex contains four subunits, including Vps23/tumor susceptibility gene 101 (TSG101), Vps28, Vps37 and multivesicular body sorting factor 12 (Mvb12) and its orthologue ubiquitin-associated protein 1 (UBAP1). TSG101 is tagged with ubiquitin chains by Rsp5, and its K63-linked ubiquitin chain is removed by DUB [77]. TSG101 is essential for ESCRT-I because of its localization and abundance. Knockdown or overexpression of TSG101 can disrupt ESCRT-I activity, which is implicated in serious developmental disorders and diseases [78]. TSG101 is usually labelled with ubiquitin by three different E3 ubiquitin ligases: mouse double minute 2 homolog (MDM2), TSG101-associated ligase (TAL), and mahogunin ring finger-1 (MGRN1). TSG101 is ubiquitinated by MDM2 and then delivered to be degraded by the proteasome [79]. However, TAL interacts with the specified domain of TSG101 and monoubiquitinates TSG101 at multiple unidentified lysine residues, leading to translocation from the membrane to the cytoplasm [80]. Alternatively, TSG101 is linked with its polyubiquitination, resulting in proteasomal degradation [81]. TSG101 is also monoubiquitinated at multiple sites by MGRN1 when vesicle trafficking is modified [82] ( Figure 3E). Although ESCRT-I ubiquitination has been well studied, the precise mechanism associated with autophagosome closure needs to be explored in the future.

### Ubiquitination regulates the critical molecular machinery of autophagosome-lysosome fusion

The final step of autophagy is the fusion of the autophagosome outer membrane with the lysosome or late endosome. The cargo within the autophagosome is released and degraded by hydrolytic enzymes. Fusion is executed by many factors, including specific N-ethylmaleimide sensitive factor attachment protein receptor (SNARE) proteins, small GTPases, motors, and ATG proteins [ 83, 84]. SNARE proteins are small integral membrane proteins containing cytoplasmic amphipathic helices. Some vesicle SNAREs form coiled-coil bundles and bridge two membranes to execute membrane fusion. Among SNAREs, syntaxin 17 localizes at the outer membrane of the completed autophagosome but not the unclosed autophagosome. It cooperates with SNAP-29 and the lysosome SNARE VAMP8 to promote autophagosome-lysosome fusion [85]. In contrast to its acetylation [86], syntaxin 17 interacts with the ubiquitin modifier BRUCE and redistributes its localization. BRUCE-deficient cells exhibit inefficient autophagosome-lysosome fusion rather than the formation of mature autophagosomes upon nutrient starvation. Therefore, syntaxin 17-positive vesicles accumulate in BRUCE-deficient cells [87]. However, BRUCE acts as a linker protein to regulate autophagy but does not exhibit ubiquitin catalytic activity.

Apart from SNARE proteins, many small GTPases serve as molecular switches and promote autophagosome-lysosome fusion. The GTPase is active after it binds to GTP when it is recruited to autophagosome membranes. Once it hydrolyses GTP to GDP, it is released from the target membrane and becomes inactive. The cycle between GTP-bound (membrane-associated) and GDP-bound (cytosolic) states is mediated by guanosine exchange factors and GTPase-activating proteins (GAPs) [88]. To date, among these small GTPases, the small GTPase Rab7 has been well characterized in autophagosome-lysosome fusion. Activated Rab7 localizes to the membrane of the autophagosome and recruits various effectors, such as motor proteins and tethering factors, to target membranes [ 89, 90]. In addition to the GTP-GDP switch, Rab7 activity is also regulated by ubiquitination. Analysis of ubiquitinated proteomes shows that there are three possible potential ubiquitinated lysine residues of Rab7, namely, K38, K191, and K126 [91]. In recent studies, the K191 residue of Rab7 has been found to be ubiquitinated, rather than K38 and K126. Site-directed mutation at K191 of Rab7 does not reduce its ubiquitination level when *TRIM39* is knocked down. TRIM39 is a member of the tripartite motif family that possesses E3 ubiquitin ligase activity. Depletion of TRIM39 suppresses autophagic flux in a Rab7 activity-dependent manner [92]. Therefore, the ubiquitination of Rab7 contributes to the fusion of autophagosomes with lysosomes. Similarly, the ubiquitinated form Ras GTPase is coimmunoprecipitated with autophagy-related proteins containing LC3 and p62. Ras GTPase is linked to K33-, K48-, and K63-polyubiquitin chains by leucine zipper-like transcriptional regulator 1 (LZTR1) and then degraded by the proteasome. LZTR1 encodes a member of the BTB-Kelch superfamily, which interacts with the Cullin3 (CUL3)-based E3 ubiquitin ligase complex [93]. Moreover, autophagosome membrane-bound Rab7 also directly interacts with its effector EPG5 and facilitates association between the Syntaxin17-SNAP29 complex and the late endosomal/lysosomal R-SNARE VAMP7/8 trans-SNAP29 complex [94]. This interaction is strengthened by the deubiquitinating enzyme USP8. USP8 binds to the CCD and directly removes nonclassical K63-linked ubiquitin chains of EPG5 at K252 [95] ( Figure 3F). Thus, ubiquitination regulates the critical molecular machinery involved in autophagosome-lysosome fusion.

## The Role of Mono- and Polyubiquitination in Selective Autophagy

Unlike nonselective bulk degradation, selective autophagy enables more specific clearance of substrates such as damaged organelles or harmful components. Hence, it plays an important role in disease prevention. To ensure specificity, the surface of these cargos is usually decorated with monoubiquitin or different polyubiquitin chains and then recognized by different autophagy receptors. These ubiquitinated cargos could be linked by autophagy receptors to the autophagosome. Because these autophagy receptors have both ubiquitin-binding domains and LC3-interacting regions (LIRs), they function as bridges to specifically recognize and recruit these cargos with ubiquitin chains [96].

**Figure 4:**
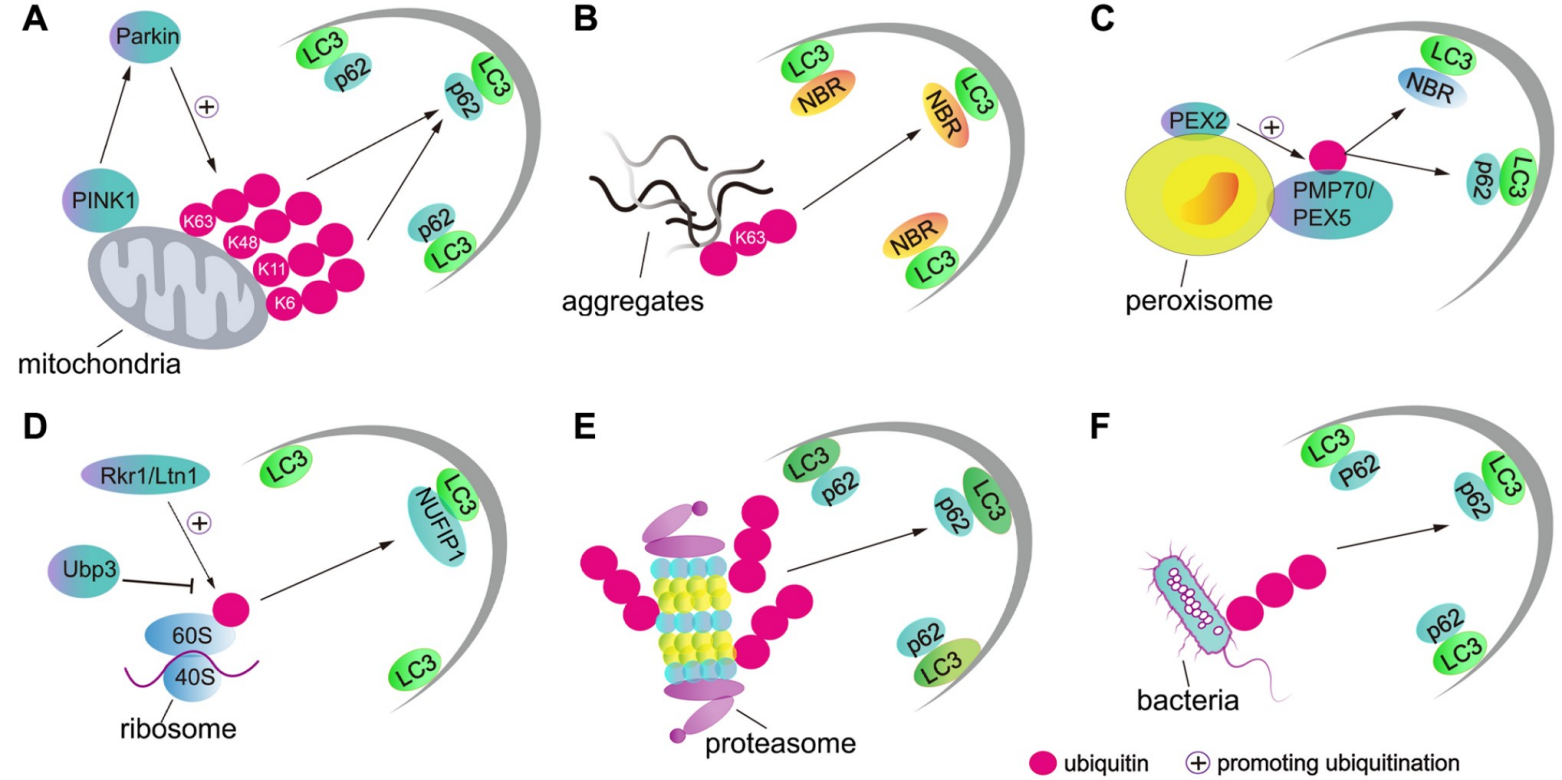
Figure 4 Ubiquitination in selective autophagy (A) During mitophagy, damaged mitochondria can be linked to the autophagy cargo receptor p62 when the ubiquitin ligase Parkin is phosphorylated to be activated by PINK1. (B) Oligomerized proteins with K63-linked ubiquitin chains are linked to p62 and then engulfed by the autophagosome; this is termed aggrephagy. (C) Peroxisome protein PMP70/PEX5 is tagged with mono-ubiquitin by the ubiquitin ligase PEX2, and this decoration is readily recognized by the autophagy cargo receptor NBR1 during pexophagy. (D) Ribosomes are recruited to the cargo receptors for degradation via autophagy after the 60s subunit of the ribosome is ubiquitinated. (E) Upon starvation, ubiquitinated proteasomes are linked to cargo receptor p62, and then interact with the autophagic membrane via the conjunct protein LC3. (F) During infection, bacteria are decorated with polyubiquitin chains and engulfed by the autophagic membrane via cargo receptor p62.

Upon mitochondrial damage, PINK1 is stabilized on the mitochondrial membrane to recruit the E3 ligase Parkin, which executes the ubiquitination of many mitochondrial outer membrane proteins [97]. Quantitative proteomic analysis also revealed that multiple types of ubiquitin chains, such as K6-, K11-, K48- and K63-linked ubiquitin chains, are generated and are Parkin-dependent when mitochondria are depolarized [ 98, 99]. Polyubiquitination of substrates is also observed in aggregates of misfolded proteins and degraded by autophagy, which is termed aggrephagy. These aggregates are usually linked to the K63-linked polyubiquitin chain rather than the K48-linked polyubiquitin chain. Subsequently, p62 bridges LC3 and ubiquitinated aggregates through its LIR domain, which facilitates selective sequestration to the autophagosome [64]. Apart from aggrephagy, autophagy receptors selectively recognize damaged peroxisomes decorated with monoubiquitination. The peroxisome membrane proteins PEX5 and PMP70 are marked with monoubiquitination by the peroxisome E3 ligase PEX2 after the peroxisome is impaired [100]. Selective autophagy has been regarded as an important way to control the quality of organelles and proteins, for example, via ribophagy and proteaphagy. The mechanism of selective autophagy is illustrated briefly in Figure 4. Although various receptors/adaptors have differing affinities for ubiquitination, it remains unclear how they recognize substrates with varied ubiquitination modifications.

## Conclusion and Perspectives

Differential ubiquitination generates structural diversity in the autophagy-critical molecular machinery that allows sequential steps of autophagy, including initiation, elongation, autophagosome maturation, and fusion. The majority of autophagy machines are decorated with K48-linked or K63-linked polyubiquitin. However, K48-linked polyubiquitination and K63-linked polyubiquitination of autophagy machines are executed by various ubiquitin ligases, and these proteins are structurally distinct. K48-linked polyubiquitination usually provides proteasomal degradation signals for autophagy machines, while K63-linked polyubiquitination represents the activation of these ATG proteins. Similarly, K11-linked polyubiquitination also yields a proteasomal degradation signal. In addition to linear polyubiquitination, mixed or branched polyubiquitination of ATG proteins has been discovered, and this type of ubiquitination is engaged in regulating the process of autophagy. These ubiquitinated ATG proteins are recognized and downregulated by UPS. Therefore, these ubiquitination modifications efficiently and effectively modulate autophagy via UPS. Furthermore, there is a regulatory feedback loop through which autophagy inhibition promotes the accumulation of ubiquitinated proteins. Autophagy inhibition leads to excessive ubiquitinated p62, which inhibits the clearance of ubiquitinated proteins destined for proteasomal degradation by delaying their delivery to the proteases of the proteasome. Thus, ubiquitination might be at the crossroads of UPS and autophagy [101].

Although ubiquitination sheds some light on the mechanism of autophagy, a key challenge is to clarify the mechanism of selectivity for mono- or polyubiquitination. In mammals, there are over 30 E2s and over 500 E3s [102]. Different combinations of E2 and E3 might result in ubiquitination versatility. Furthermore, many substrates can be ubiquitinated on one or more of their lysine residues. E3 ligases prefer a specific lysine residue and determine the lysine section. Moreover, before ubiquitination, other posttranscriptional modifications of substrates or ubiquitin, for example, phosphorylation, are important in determining whether mono- or polyubiquitination occurs because these modifications result in the substrates generating specific structures and have an important impact on ubiquitination modification. Furthermore, the proteins that interact with the substrates also affect the selectivity between mono- and polyubiquitination. In addition to these factors, ubiquitin itself also determines mono- and polyubiquitination because of posttranslational modifications or the binding of small molecules on ubiquitin [ 7, 103, 104 ]. These possibilities need to be clarified and explained by high-resolution imaging with the help of structural biology approaches in the future.
